# (*E*)-1-(3,5-Di­meth­oxy­phen­yl)-3-(3-meth­oxy­phen­yl)prop-2-en-1-one

**DOI:** 10.1107/S1600536813008982

**Published:** 2013-04-05

**Authors:** Seunghyun Ahn, Ha-Jin Lee, Yoongho Lim, Dongsoo Koh

**Affiliations:** a Department of Applied Chemistry, Dongduk Women’s University, Seoul 136-714, Republic of Korea; b Jeonju Center, Korea Basic Science Center (KBSI), Jeonju 561-765, Republic of Korea; c Division of Bioscience and Biotechnology, BMIC, Konkuk University, Seoul 143-701, Republic of Korea

## Abstract

In the title mol­ecule, C_18_H_18_O_4_, the C=C bond of the central enone group adopts a *trans* conformation. The relative conformation of the C=O and C=C bonds is *s*-*cisoid*. The dihedral angle between the planes of the benzene rings is 29.49 (12)°. In the crystal, weak C—H⋯O hydrogen bonds link the mol­ecules into chains along [010].

## Related literature

For the synthesis and biological properties of chalcone derivatives, see: Shenvi *et al.* (2013 ▶); Hsieh *et al.* (2012 ▶); Hwang *et al.* (2011 ▶); Jo *et al.* (2012 ▶); Sharma *et al.* (2012 ▶); Sashidhara *et al.* (2011 ▶). For related structures, see: Carvalho-Jr *et al.* (2011 ▶); Wu *et al.* (2012 ▶).
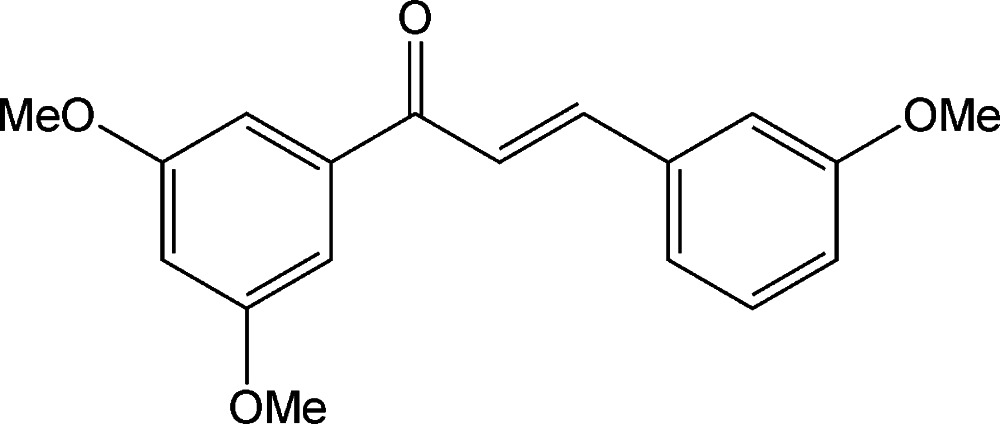


## Experimental

### 

#### Crystal data

C_18_H_18_O_4_
*M* *_r_* = 298.32
Triclinic, 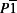

*a* = 8.2402 (12) Å
*b* = 9.1449 (14) Å
*c* = 10.8876 (16) Å
α = 95.395 (3)°
β = 107.667 (3)°
γ = 102.837 (3)°
*V* = 750.61 (19) Å^3^
*Z* = 2
Mo *K*α radiation
μ = 0.09 mm^−1^
*T* = 200 K
0.33 × 0.26 × 0.12 mm

#### Data collection

Bruker SMART CCD diffractometer
Absorption correction: multi-scan (*SADABS*; Bruker, 2000 ▶) *T*_min_ = 0.970, *T*_max_ = 0.989
4383 measured reflections
2619 independent reflections
1531 reflections with *I* > 2σ(*I*)
*R*_int_ = 0.028

#### Refinement

*R*[*F* ^2^ > 2σ(*F* ^2^)] = 0.044
*wR*(*F* ^2^) = 0.115
*S* = 0.98
2619 reflections
202 parameters
H-atom parameters constrained
Δρ_max_ = 0.20 e Å^−3^
Δρ_min_ = −0.26 e Å^−3^

### 

Data collection: *SMART* (Bruker, 2000 ▶); cell refinement: *SAINT* (Bruker, 2000 ▶); data reduction: *SAINT*; program(s) used to solve structure: *SHELXTL* (Sheldrick, 2008 ▶); program(s) used to refine structure: *SHELXTL*; molecular graphics: *PLATON* (Spek, 2009 ▶); software used to prepare material for publication: *SHELXTL*.

## Supplementary Material

Crystal structure: contains datablock(s) I, global. DOI: 10.1107/S1600536813008982/lh5590sup1.cif

Structure factors: contains datablock(s) I. DOI: 10.1107/S1600536813008982/lh5590Isup2.hkl

Supplementary material file. DOI: 10.1107/S1600536813008982/lh5590Isup3.cml

Additional supplementary materials:  crystallographic information; 3D view; checkCIF report

## Figures and Tables

**Table 1:** Hydrogen-bond geometry (Å, °)

*D*—H⋯*A*	*D*—H	H⋯*A*	*D*⋯*A*	*D*—H⋯*A*
C7—H7⋯O4^i^	0.95	2.55	3.405 (3)	151
C9—H9*C*⋯O1^ii^	0.98	2.50	3.388 (3)	150

Symmetry codes: (i) 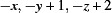; (ii) 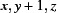.

**Fig. 1.**
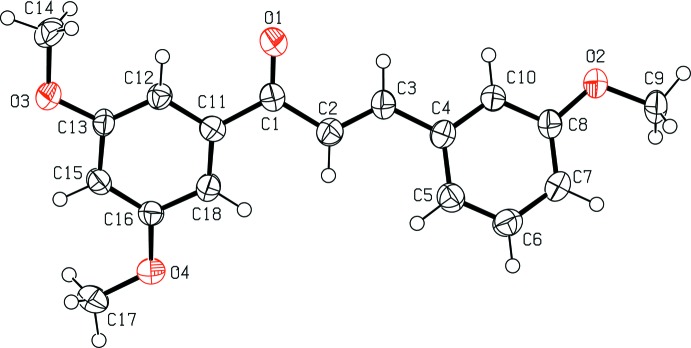
The molecular structure of the title compound, showing displacement ellipsoids drawn at the 50% probability level.

**Fig. 2.**
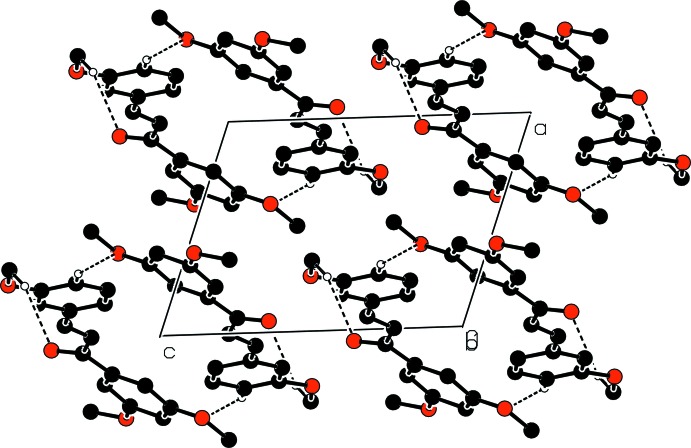
Part of the crystal structure with weak intermolecular C—H···O hydrogen bonds shown as dashed lines.

## supplementary crystallographic information

### Comment

Chalcones are one of the secondary metabolites in plants and belong to a flavonoid class. They have shown diverse biological activities including anti-cancer (Shenvi *et al.* 2013), anti-microbial (Sharma *et al.* 2012), anti-diabetic (Hsieh *et al.* 2012) and anti-inflammatory (Sashidhara *et al.* 2011). As a part of our studies on the substituent effects of chalcones on structures and biological activities (Jo *et al.*, 2012; Hwang *et al.*, 2011), the title compound was synthesized and its crystal structure was determined.

The molecular structure of the title compound is shown in Fig. 1. The *trans* configuration of C2═C3 double bond is defined by the dihedral angle of -177.9 (2) Å for C1—C2—C3—C4. The relative conformation of two double bonds, O1═C1 and C2═C3, is s-*cisoid* with a torsion angle of -12.8 (4)° for O1—C1—C2—C3. The orientations of the three methoxy groups can be defined by the torsion angles C9—O2—C8—C10 [177.1 (3)°], C17—O4—C16—C15 [-1.8 (5)°] and C14—O3—C13—C15 [-172.2 (3)°]. The dihedral angle between the benzene rings is 29.49 (12)°. In the crystal, weak C—H···O hydrogen bonds links the molecules into chains along [010] (Fig. 2). Some examples of methoxy substituted chalcone structures have been published (Wu *et al.*, 2012; Carvalho-Jr *et al.*, 2011).

### Experimental

To a solution of 3-methoxybenzaldehyde (136 mg, 1 mmol) in 20 ml of ethanol was added 3,5-dimethoxyacetophenone (180 mg, 1 mmol) and the temperature was adjusted to around 276 K in an ice-bath. To the cooled reaction mixture was added 1 ml of 50% aqueous KOH solution, and the reaction mixture was stirred at room temperature for 20 h. This mixture was poured into iced water (30 ml) was acidified (pH =3) with 3 N HCl solution to give a precipitate. Filtration and washing with water afforded crude solid of the title compound (240 mg, 79%). Recrystallization of the solid in ethanol gave pale yellow crystals which were suitable for X-ray diffraction (mp: 363–364 K).

### Refinement

H atoms were placed in calculated positions and refined as riding with C—H = 0.95–0.98 Å, and *U*_iso_(H) = 1.2 *U*_eq_(C) or *U*_iso_(H) = 1.5*U*_eq_(C_methyl_). Four outliers were removed in the final refinement (4 2 8, -7 5 9, -7, -3, 10 and -7, -4, 9).

### Crystal data

**Table:**

C_18_H_18_O_4_	*Z* = 2
*M**_r_* = 298.32	*F*(000) = 316
Triclinic, *P*1	*D*_x_ = 1.320 Mg m^−^^3^
Hall symbol: -P 1	Mo *K*α radiation, λ = 0.71073 Å
*a* = 8.2402 (12) Å	Cell parameters from 1553 reflections
*b* = 9.1449 (14) Å	θ = 2.3–27.9°
*c* = 10.8876 (16) Å	µ = 0.09 mm^−^^1^
α = 95.395 (3)°	*T* = 200 K
β = 107.667 (3)°	Block, pale yellow
γ = 102.837 (3)°	0.33 × 0.26 × 0.12 mm
*V* = 750.61 (19) Å^3^	

### Data collection

**Table:**

Bruker SMART CCD diffractometer	2619 independent reflections
Radiation source: fine-focus sealed tube	1531 reflections with *I* > 2σ(*I*)
Graphite monochromator	*R*_int_ = 0.028
φ and ω scans	θ_max_ = 25.0°, θ_min_ = 2.0°
Absorption correction: multi-scan (*SADABS*; Bruker, 2000)	*h* = −7→9
*T*_min_ = 0.970, *T*_max_ = 0.989	*k* = −9→10
4383 measured reflections	*l* = −12→12

### Refinement

**Table:**

Refinement on *F*^2^	Primary atom site location: structure-invariant direct methods
Least-squares matrix: full	Secondary atom site location: difference Fourier map
*R*[*F*^2^ > 2σ(*F*^2^)] = 0.044	Hydrogen site location: inferred from neighbouring sites
*wR*(*F*^2^) = 0.115	H-atom parameters constrained
*S* = 0.98	*w* = 1/[σ^2^(*F*_o_^2^) + (0.0394*P*)^2^] where *P* = (*F*_o_^2^ + 2*F*_c_^2^)/3
2619 reflections	(Δ/σ)_max_ < 0.001
202 parameters	Δρ_max_ = 0.20 e Å^−^^3^
0 restraints	Δρ_min_ = −0.26 e Å^−^^3^

### Special details

**Table:**

Geometry. All e.s.d.'s (except the e.s.d. in the dihedral angle between two l.s. planes) are estimated using the full covariance matrix. The cell e.s.d.'s are taken into account individually in the estimation of e.s.d.'s in distances, angles and torsion angles; correlations between e.s.d.'s in cell parameters are only used when they are defined by crystal symmetry. An approximate (isotropic) treatment of cell e.s.d.'s is used for estimating e.s.d.'s involving l.s. planes.
Refinement. Refinement of *F*^2^ against ALL reflections. The weighted *R*-factor *wR* and goodness of fit *S* are based on *F*^2^, conventional *R*-factors *R* are based on *F*, with *F* set to zero for negative *F*^2^. The threshold expression of *F*^2^ > σ(*F*^2^) is used only for calculating *R*-factors(gt) *etc*. and is not relevant to the choice of reflections for refinement. *R*-factors based on *F*^2^ are statistically about twice as large as those based on *F*, and *R*- factors based on ALL data will be even larger.

### Fractional atomic coordinates and isotropic or equivalent isotropic displacement parameters (Å^2^)

**Table:**

	*x*	*y*	*z*	*U*_iso_*/*U*_eq_	
C1	0.0722 (3)	0.0058 (3)	0.7610 (2)	0.0354 (6)	
O1	0.0527 (2)	−0.06317 (18)	0.65288 (16)	0.0503 (5)	
C2	−0.0008 (3)	0.1386 (3)	0.7724 (2)	0.0385 (6)	
H2	−0.0059	0.1771	0.8549	0.046*	
C3	−0.0595 (3)	0.2054 (3)	0.6708 (2)	0.0355 (6)	
H3	−0.0560	0.1615	0.5893	0.043*	
C4	−0.1290 (3)	0.3399 (2)	0.6711 (2)	0.0338 (6)	
C5	−0.1565 (3)	0.4081 (3)	0.7811 (2)	0.0413 (6)	
H5	−0.1313	0.3665	0.8594	0.050*	
C6	−0.2199 (3)	0.5353 (3)	0.7760 (2)	0.0435 (7)	
H6	−0.2397	0.5798	0.8508	0.052*	
C7	−0.2555 (3)	0.6000 (3)	0.6635 (2)	0.0407 (6)	
H7	−0.2990	0.6879	0.6611	0.049*	
C8	−0.2268 (3)	0.5343 (3)	0.5553 (2)	0.0359 (6)	
O2	−0.2555 (2)	0.58787 (18)	0.43929 (15)	0.0456 (5)	
C9	−0.3268 (3)	0.7168 (3)	0.4271 (2)	0.0462 (7)	
H9A	−0.4436	0.6916	0.4371	0.069*	
H9B	−0.3375	0.7444	0.3407	0.069*	
H9C	−0.2481	0.8028	0.4952	0.069*	
C10	−0.1656 (3)	0.4048 (2)	0.5588 (2)	0.0341 (6)	
H10	−0.1485	0.3597	0.4831	0.041*	
C11	0.1748 (3)	−0.0419 (3)	0.8812 (2)	0.0325 (6)	
C12	0.2212 (3)	−0.1790 (2)	0.8674 (2)	0.0323 (6)	
H12	0.1800	−0.2429	0.7842	0.039*	
C13	0.3267 (3)	−0.2205 (2)	0.9747 (2)	0.0329 (6)	
O3	0.3825 (2)	−0.35105 (17)	0.97449 (15)	0.0466 (5)	
C14	0.3448 (4)	−0.4415 (3)	0.8493 (2)	0.0493 (7)	
H14A	0.2167	−0.4794	0.8067	0.074*	
H14B	0.3970	−0.5278	0.8611	0.074*	
H14C	0.3951	−0.3791	0.7947	0.074*	
C15	0.3874 (3)	−0.1279 (2)	1.0980 (2)	0.0342 (6)	
H15	0.4604	−0.1577	1.1718	0.041*	
C16	0.3407 (3)	0.0062 (3)	1.1115 (2)	0.0321 (6)	
O4	0.3951 (2)	0.10519 (17)	1.22797 (15)	0.0441 (5)	
C17	0.5018 (4)	0.0614 (3)	1.3408 (2)	0.0491 (7)	
H17A	0.4375	−0.0362	1.3542	0.074*	
H17B	0.5295	0.1393	1.4177	0.074*	
H17C	0.6116	0.0510	1.3279	0.074*	
C18	0.2335 (3)	0.0503 (3)	1.0033 (2)	0.0349 (6)	
H18	0.2008	0.1428	1.0131	0.042*	

### Atomic displacement parameters (Å^2^)

**Table:**

	*U*^11^	*U*^22^	*U*^33^	*U*^12^	*U*^13^	*U*^23^
C1	0.0339 (15)	0.0363 (14)	0.0369 (15)	0.0102 (12)	0.0112 (13)	0.0105 (12)
O1	0.0648 (14)	0.0511 (11)	0.0357 (11)	0.0269 (10)	0.0094 (10)	0.0079 (9)
C2	0.0385 (16)	0.0407 (15)	0.0398 (15)	0.0142 (13)	0.0146 (13)	0.0088 (12)
C3	0.0323 (15)	0.0366 (14)	0.0396 (15)	0.0115 (12)	0.0115 (13)	0.0117 (11)
C4	0.0272 (14)	0.0336 (14)	0.0406 (15)	0.0078 (11)	0.0114 (12)	0.0069 (11)
C5	0.0418 (16)	0.0444 (15)	0.0401 (15)	0.0131 (13)	0.0146 (14)	0.0118 (12)
C6	0.0485 (18)	0.0438 (16)	0.0443 (16)	0.0163 (14)	0.0213 (14)	0.0069 (13)
C7	0.0427 (17)	0.0354 (15)	0.0482 (16)	0.0174 (13)	0.0158 (14)	0.0078 (12)
C8	0.0330 (15)	0.0375 (15)	0.0388 (15)	0.0129 (12)	0.0102 (13)	0.0113 (12)
O2	0.0589 (13)	0.0466 (11)	0.0424 (11)	0.0297 (9)	0.0186 (10)	0.0166 (8)
C9	0.0516 (18)	0.0417 (16)	0.0501 (17)	0.0249 (14)	0.0123 (15)	0.0168 (13)
C10	0.0353 (15)	0.0354 (14)	0.0342 (14)	0.0126 (12)	0.0123 (12)	0.0081 (11)
C11	0.0292 (14)	0.0365 (14)	0.0313 (14)	0.0071 (11)	0.0101 (12)	0.0063 (11)
C12	0.0325 (14)	0.0287 (13)	0.0342 (14)	0.0077 (11)	0.0096 (12)	0.0040 (11)
C13	0.0361 (15)	0.0306 (13)	0.0360 (14)	0.0148 (12)	0.0117 (12)	0.0110 (11)
O3	0.0637 (13)	0.0386 (10)	0.0405 (10)	0.0267 (10)	0.0127 (10)	0.0056 (8)
C14	0.0572 (19)	0.0384 (15)	0.0497 (17)	0.0190 (14)	0.0129 (15)	−0.0035 (13)
C15	0.0348 (15)	0.0396 (15)	0.0300 (14)	0.0115 (12)	0.0107 (12)	0.0112 (11)
C16	0.0325 (14)	0.0352 (14)	0.0297 (13)	0.0101 (12)	0.0110 (12)	0.0051 (11)
O4	0.0544 (12)	0.0433 (10)	0.0340 (10)	0.0195 (9)	0.0105 (9)	0.0016 (8)
C17	0.0529 (18)	0.0585 (18)	0.0314 (15)	0.0178 (15)	0.0069 (14)	0.0031 (13)
C18	0.0347 (15)	0.0342 (14)	0.0412 (15)	0.0135 (12)	0.0158 (13)	0.0113 (12)

### Geometric parameters (Å, º)

**Table:**

C1—O1	1.229 (2)	C10—H10	0.9500
C1—C2	1.481 (3)	C11—C18	1.390 (3)
C1—C11	1.489 (3)	C11—C12	1.397 (3)
C2—C3	1.329 (3)	C12—C13	1.371 (3)
C2—H2	0.9500	C12—H12	0.9500
C3—C4	1.467 (3)	C13—O3	1.371 (2)
C3—H3	0.9500	C13—C15	1.400 (3)
C4—C10	1.389 (3)	O3—C14	1.433 (2)
C4—C5	1.401 (3)	C14—H14A	0.9800
C5—C6	1.376 (3)	C14—H14B	0.9800
C5—H5	0.9500	C14—H14C	0.9800
C6—C7	1.390 (3)	C15—C16	1.373 (3)
C6—H6	0.9500	C15—H15	0.9500
C7—C8	1.379 (3)	C16—O4	1.373 (2)
C7—H7	0.9500	C16—C18	1.395 (3)
C8—O2	1.370 (2)	O4—C17	1.426 (3)
C8—C10	1.385 (3)	C17—H17A	0.9800
O2—C9	1.429 (2)	C17—H17B	0.9800
C9—H9A	0.9800	C17—H17C	0.9800
C9—H9B	0.9800	C18—H18	0.9500
C9—H9C	0.9800		
			
O1—C1—C2	120.5 (2)	C4—C10—H10	119.4
O1—C1—C11	119.6 (2)	C18—C11—C12	120.0 (2)
C2—C1—C11	119.9 (2)	C18—C11—C1	121.7 (2)
C3—C2—C1	122.0 (2)	C12—C11—C1	118.1 (2)
C3—C2—H2	119.0	C13—C12—C11	119.5 (2)
C1—C2—H2	119.0	C13—C12—H12	120.2
C2—C3—C4	126.9 (2)	C11—C12—H12	120.2
C2—C3—H3	116.6	C12—C13—O3	125.4 (2)
C4—C3—H3	116.6	C12—C13—C15	120.8 (2)
C10—C4—C5	118.3 (2)	O3—C13—C15	113.8 (2)
C10—C4—C3	118.9 (2)	C13—O3—C14	116.83 (18)
C5—C4—C3	122.7 (2)	O3—C14—H14A	109.5
C6—C5—C4	120.1 (2)	O3—C14—H14B	109.5
C6—C5—H5	120.0	H14A—C14—H14B	109.5
C4—C5—H5	120.0	O3—C14—H14C	109.5
C5—C6—C7	121.2 (2)	H14A—C14—H14C	109.5
C5—C6—H6	119.4	H14B—C14—H14C	109.5
C7—C6—H6	119.4	C16—C15—C13	119.6 (2)
C8—C7—C6	118.9 (2)	C16—C15—H15	120.2
C8—C7—H7	120.5	C13—C15—H15	120.2
C6—C7—H7	120.5	C15—C16—O4	123.8 (2)
O2—C8—C7	124.4 (2)	C15—C16—C18	120.3 (2)
O2—C8—C10	115.29 (19)	O4—C16—C18	115.8 (2)
C7—C8—C10	120.3 (2)	C16—O4—C17	117.02 (18)
C8—O2—C9	118.08 (17)	O4—C17—H17A	109.5
O2—C9—H9A	109.5	O4—C17—H17B	109.5
O2—C9—H9B	109.5	H17A—C17—H17B	109.5
H9A—C9—H9B	109.5	O4—C17—H17C	109.5
O2—C9—H9C	109.5	H17A—C17—H17C	109.5
H9A—C9—H9C	109.5	H17B—C17—H17C	109.5
H9B—C9—H9C	109.5	C11—C18—C16	119.7 (2)
C8—C10—C4	121.2 (2)	C11—C18—H18	120.2
C8—C10—H10	119.4	C16—C18—H18	120.2
			
O1—C1—C2—C3	−12.8 (4)	O1—C1—C11—C12	−10.3 (3)
C11—C1—C2—C3	165.3 (2)	C2—C1—C11—C12	171.6 (2)
C1—C2—C3—C4	−177.9 (2)	C18—C11—C12—C13	−0.8 (3)
C2—C3—C4—C10	172.7 (2)	C1—C11—C12—C13	175.2 (2)
C2—C3—C4—C5	−5.9 (4)	C11—C12—C13—O3	−179.6 (2)
C10—C4—C5—C6	0.6 (4)	C11—C12—C13—C15	0.4 (3)
C3—C4—C5—C6	179.2 (2)	C12—C13—O3—C14	7.9 (3)
C4—C5—C6—C7	−0.9 (4)	C15—C13—O3—C14	−172.10 (19)
C5—C6—C7—C8	0.2 (4)	C12—C13—C15—C16	0.0 (3)
C6—C7—C8—O2	−179.5 (2)	O3—C13—C15—C16	−180.0 (2)
C6—C7—C8—C10	0.9 (4)	C13—C15—C16—O4	−179.33 (19)
C7—C8—O2—C9	−2.6 (4)	C13—C15—C16—C18	0.0 (3)
C10—C8—O2—C9	177.0 (2)	C15—C16—O4—C17	−2.0 (3)
O2—C8—C10—C4	179.2 (2)	C18—C16—O4—C17	178.7 (2)
C7—C8—C10—C4	−1.2 (4)	C12—C11—C18—C16	0.8 (3)
C5—C4—C10—C8	0.4 (4)	C1—C11—C18—C16	−175.1 (2)
C3—C4—C10—C8	−178.2 (2)	C15—C16—C18—C11	−0.4 (3)
O1—C1—C11—C18	165.7 (2)	O4—C16—C18—C11	178.96 (19)
C2—C1—C11—C18	−12.4 (3)		

### Hydrogen-bond geometry (Å, º)

**Table:**

*D*—H···*A*	*D*—H	H···*A*	*D*···*A*	*D*—H···*A*
C7—H7···O4^i^	0.95	2.55	3.405 (3)	151
C9—H9*C*···O1^ii^	0.98	2.50	3.388 (3)	150

Symmetry codes: (i) −*x*, −*y*+1, −*z*+2; (ii) *x*, *y*+1, *z*.
